# Deciphering Anticancer Mechanisms of Calycosin in Lung Adenocarcinoma Through Multi-Omics: Targeting SMAD3-Mediated NOTCH Signaling in the Tumor Microenvironment

**DOI:** 10.3390/cancers17091455

**Published:** 2025-04-26

**Authors:** Bi-Tian Zhang, Xiaoyu Song, Chi-Shing (William) Cho, Chun-Kwok Wong, Dongjie Wang

**Affiliations:** 1 Institute of Chinese Medicine and State Key Laboratory of Research on Bioactivities and Clinical Applications of Medicinal Plants, The Chinese University of Hong Kong, Hong Kong, China; bitianzhang@link.cuhk.edu.hk; 2 School of Life Science and Technology, Harbin Institute of Technology, Harbin 150001, China; songxyhit@hit.edu.cn; 3 Department of Clinical Oncology, Queen Elizabeth Hospital, Kowloon, Hong Kong, China; williamcscho@gmail.com; 4 Department of Chemical Pathology, The Chinese University of Hong Kong, Prince of Wales Hospital, Hong Kong, China; 5 Li Dak Sum Yip Yio Chin R & D Centre for Chinese Medicine, The Chinese University of Hong Kong, Hong Kong, China; 6 Shenzhen Research Institute, The Chinese University of Hong Kong, Shenzhen 518172, China

**Keywords:** calycosin, SMAD3, NOTCH signaling, monocytes, macrophages, LUAD, KMT2A

## Abstract

Objective: Lung adenocarcinoma (LUAD) remains a leading cause of cancer-related mortality, particularly in advanced stages. This study investigates the anticancer mechanisms of calycosin, an isoflavonoid derived from *Astragalus membranaceus*, in LUAD. Methods: Using integrative approaches including bulk and single-cell RNA sequencing, network pharmacology, and molecular docking, we identified SMAD3 as a critical biomarker associated with LUAD staging and prognosis. Results: Calycosin targets SMAD3, modulating the NOTCH signaling pathway in monocytes/macrophages to suppress tumor growth, invasion, and immune evasion. Enrichment analyses revealed significant involvement of NOTCH signaling components in SMAD3-correlated genes, particularly in advanced-stage LUAD. Single-cell RNA sequencing further demonstrated NOTCH pathway enrichment in tumor-associated monocytes/macrophages. Additionally, KMT2A was identified as a key transcriptional regulator in these cells. Conclusions: These findings highlight the potential effects of calycosin and provide novel insights into targeting the tumor–immune microenvironment in LUAD.

## Simple Summary

Lung adenocarcinoma (LUAD) is a leading cause of cancer deaths, particularly in advanced stages. This study investigates calycosin, a compound from *Astragalus membranaceus*, for its potential to combat LUAD by targeting SMAD3 and modulating the NOTCH signaling pathway in monocytes/macrophages. These immune cells are crucial in promoting tumor growth and helping cancers evade the immune system. By disrupting harmful processes driven by SMAD3 and NOTCH signaling, calycosin can reduce cancer progression. Additionally, KMT2A serves as a key transcriptional regulator in monocytes/macrophages, suggesting its role in coordinating NOTCH-dependent transcriptional programs. This highlights new opportunities for targeting myeloid cell functions in LUAD. These findings could lead to innovative therapies that improve patient outcomes by reshaping the environment around tumors.

## 1. Introduction

Lung adenocarcinoma (LUAD), a predominant subtype of non-small-cell lung cancer (NSCLC), accounts for approximately 40% of all lung cancer cases and remains a leading cause of cancer-related mortality worldwide [1,2]. Despite advances in surgical intervention, chemotherapy, targeted therapies, and immunotherapy, the prognosis for patients with advanced-stage LUAD (stages III and IV) remains poor, with a 5-year survival rate below 20% [3]. In contrast, early-stage LUAD (stages I and II) exhibits more localized disease patterns, often confined to the lung parenchyma without extensive lymph node involvement or distant metastasis, resulting in significantly higher survival rates when treated promptly [4]. However, the molecular mechanisms underlying the progression from early to advanced stages remain incompletely understood, underscoring the urgent need for novel strategies targeting the key drivers of LUAD progression [5].

SMAD3, a key mediator of the TGF-β signaling pathway [6], demonstrates significant upregulation in advanced-stage LUAD compared with early-stage disease, correlating with increased tumor aggressiveness, invasive capacity, and poor patient outcomes [7]. This stage-specific expression pattern highlights the potential of SMAD3 as a diagnostic tool [8], warranting further investigation into its regulatory networks and interactions within the tumor microenvironment.

Traditional Chinese medicine (TCM) has long been explored as a source of bioactive compounds with potential anticancer properties [9]. Among these, *Astragalus membranaceus*, a widely used medicinal herb in TCM, has garnered considerable attention due to its reported immunomodulatory, anti-inflammatory [10], and antitumor effects [11]. One of the major bioactive flavonoids isolated from *Astragalus*, calycosin [12], has emerged as a promising candidate for cancer therapy [13]. Calycosin exhibits diverse pharmacological activities, including antioxidant, anti-inflammatory, and estrogenic effects, and has shown preliminary efficacy in suppressing tumor growth in various cancer models [12,14,15]. Our findings reveal that calycosin exerts its anticancer effects by acting on SMAD3 through the NOTCH signaling pathway in monocytes/macrophages, highlighting the intricate interplay between tumor cells and the immune microenvironment in LUAD pathogenesis.

Recent studies have highlighted the critical role of aberrant signaling pathways and metabolic reprogramming in LUAD progression. For instance, the activation of oncogenic pathways such as PI3K/AKT/mTOR and MAPK/ERK, alongside the dysregulation of tumor suppressor genes like TP53, contributes to the aggressive behavior of high-stage LUAD [16]. Based on our previous studies, the PI3K/AKT signaling pathway has a significant role in tumorigenesis such as lung cancer development [17,18]. Furthermore, advanced-stage tumors are characterized by enhanced epithelial–mesenchymal transition (EMT), increased angiogenesis, and resistance to apoptosis, enabling their invasive and metastatic capabilities [19]. This EMT process is regulated by both the PI3K/AKT and NOTCH signaling pathways [20]. In contrast, low-stage LUAD tumors typically exhibit less aggressive phenotypes with slower proliferation rates and limited metastatic potential [21]. These distinctions underscore the importance of identifying stage-specific targets to improve patient outcomes.

The tumor microenvironment (TME), particularly the interaction between tumor cells and immune cells such as monocytes/macrophages, plays a crucial role in LUAD progression [22]. Monocytes/macrophages are known to promote tumor growth, invasion, and immune evasion through the activation of key signaling pathways [23], including NOTCH [24]. Our study reveals that calycosin disrupts this process by targeting SMAD3 and modulating NOTCH signaling in monocytes/macrophages, thereby inhibiting tumor-supportive functions and reversing the immunosuppressive TME. This novel mechanism not only underscores the potential effects of calycosin but also provides a deeper understanding of the crosstalk between SMAD3, NOTCH signaling, and the immune microenvironment in LUAD.

In this study, we investigated the anticancer mechanisms of calycosin in LUAD, focusing on its ability to modulate the key signaling pathways implicated in tumor progression. Our findings reveal that calycosin exerts potent inhibitory effects on LUAD cell proliferation, migration, and invasion while simultaneously inducing apoptosis and reversing EMT. Notably, these effects were more pronounced in advanced-stage LUAD models, suggesting that calycosin may serve as a stage-specific anticancer agent. By elucidating the molecular interplay between calycosin and LUAD pathogenesis, our work not only highlights the potential effects of *Astragalus*-derived compounds but also provides novel insights into the development of targeted therapies for advanced-stage LUAD.

## 2. Methods

### 2.1. Data Collection

In this study, the bulk RNA sequencing datasets of lung adenocarcinoma (LUAD) were collected from The Cancer Genome Atlas (TCGA) Program Database (https://www.cancer.gov/ccg/research/genome-sequencing/tcga) (accessed on 4 March 2025), and they include the gene expression and clinical data of 436 patients. Additionally, the single-cell RNA sequencing dataset of breast cancer was sourced from The Gene Expression Omnibus Database (GEO) (https://www.ncbi.nlm.nih.gov/geo/) (accessed on 15 March 2025) with the serial number GSE99254. The drug targets of the calycosin were downloaded from the BATMAN-TCM Database (http://bionet.ncpsb.org.cn/batman-tcm/index.php) (accessed on 4 March 2025). Furthermore, the genes involved in NOTCH signaling pathway were searched from the Gene Set Enrichment Analysis Database (GSEA) (https://www.gsea-msigdb.org/gsea/index.jsp) (accessed on 5 March 2025).

### 2.2. Liquid Chromatography–Mass Spectrometry

In order to analyze the anticancer mechanism of the *Astragalus membranaceus* compound calycosin, first, we used liquid chromatography to elute compounds from the herb, and then we performed the mass spectrometry to quantify the detailed compound compositions.

### 2.3. Network Pharmacology

In order to investigate the interactions between the disease targets of LUAD and calycosin in the traditional Chinese medicine *Astragalus membranaceus*, the method of network pharmacology was applied for analyzing the underlying molecular mechanism [25].

### 2.4. Immunohistochemical Staining

Immunohistochemical staining is a method generally used to show protein expression in tissues. We used IHC images from the Human Protein Atlas (HPA) database (https://www.proteinatlas.org) (accessed on 4 March 2025), a publicly available resource that provides high-quality IHC-stained tissue images for various proteins and tissues. The HPA database employs standardized protocols for tissue processing, staining, and imaging, ensuring consistency and reliability in the data. We analyzed normal tissue samples and tumor tissue samples of lung adenocarcinoma (as documented in the HPA database) [26] to compare the protein expression levels of SMAD3.

### 2.5. Prognostic Model Construction

The genes that exert major roles in LUAD that are linked with the cancer stages influencing patients’ survival were screened using Least Absolute Shrinkage and Selection Operator (LASSO) Cox regression. Usually, there might be multiple genes after the screening of LASSO-Cox regression, and other measures such as the Multivariate Survival Regression and Kaplan–Meier (KM) curve were also used to further select the genes that are most correlated with patients’ prognosis affected by clinical stage in LUAD. Accuracy of the prognostic model was verified using Receiver Operating Characteristic (ROC) curves [27]. This was performed with R (Version 4.4.0).

### 2.6. Molecular Docking Analysis for SMAD3-Drug Interactions

After identifying SMAD3 as a potential biomarker for cancer stages in LUAD, we proceeded to evaluated the virtual binding affinity and possibility between drug molecules and active binding site of the SMAD3 protein using molecular docking techniques. In the docking process, we first downloaded the spatial structures of SMAD3 protein and small molecule calycosin. Then, through the online platform CB-DOCK2 (https://cadd.labshare.cn/) (accessed on 4 March 2025), the structures of the protein and calycosin were input to define the coordinates of possible binding pockets. Calycosin was docked into the binding pocket, and the docking scores were calculated. Usually, a docking score below −5 kcal/mol is preferred for binding between molecules and binding sites [28].

### 2.7. Pearson’s Correlation Analysis to Assess Gene–Gene Relationships in LUAD

To ascertain the correlation of various genes with the SMAD3 gene within the LUAD dataset, we employed the Pearson’s correlation analysis. This analysis was conducted using R (Version 4.4.0), which facilitated the computation of the correlation coefficient. The resulting values, ranging from −1 to 1, indicated the strength and direction of the correlation: a positive value denoted a direct correlation, while a negative value indicated an inverse relationship. Concurrently, we generated *p*-values to assess the statistical significance of these correlations. The correlations with *p*-values lower than 0.05 were considered to indicate a significant correlation, thereby helping to discern meaningful associations between genes and SMAD3 within the context of LUAD. This methodological approach ensured a robust statistical framework for understanding the interplay of genes in LUAD biology, particularly in relation to SMAD3.

### 2.8. Weight Correlation Network Analysis for SMAD3-Related Genes in LUAD

To substantiate the role of SMAD3-related genes in the regulation of the NOTCH signaling pathway in LUAD, we employed the method of Weight Correlation Network Analysis (WGCNA). Initially we calculated the Pearson’s correlation coefficient for all genes in relation to SMAD3. Subsequently, we utilized the Limma algorithm to ascertain the statistical significance of genes within the LUAD dataset, which was sourced from the TCGA online repository. Following this, we identified a set of genes that were both correlated with SMAD3 based on Pearson’s correlation and with significance according to the Limma algorithm. These genes were then subjected to WGCNA analysis to explore their co-expression patterns. The genes were categorized into distinct modules predicated on their co-expression profiles. Specifically, those co-expressed with SMAD3 were further analyzed to elucidate their potential mechanisms in the pathogenesis of LUAD.

### 2.9. Enrichment Analysis

For the enrichment analysis, the gene annotation was accessed through KEGG rest API (https://www.kegg.jp/kegg/rest/keggapi.html) (accessed on 5 March 2025). Then, the “ClusterProfiler” R package (R version 4.4.0) was utilized to perform the analysis. The smallest gene set was set as 5 genes, and the largest was set as 5000 genes. The critical value showing statistical significance was set as 0.05. Here, the enrichment analysis consisted of Gene Ontology (GO) enrichment, Kyoto Encyclopedia of Genes and Genomes (KEGG) enrichment, and Gene Set Enrichment analysis. The GO enrichment analysis was further divided into Biological Process (BP), Cellular Component (CC), and Molecular Function (MF) dimensions.

### 2.10. Differential Immune Cell Infiltration Analysis of SMAD3

Following the screening of the prognostic marker for LUAD, the correlation between the SMAD3 and other immune checkpoint genes in LUAD were plotted in heatmap. R (Version 4.4.0) was used to calculate the correlation between the expression of SMAD3 and immune cell infiltration scores in LUAD dataset. Then, the dot plot was plotted in R (Version 4.4.0). The correlation between the key gene expression and cell infiltration score was plotted using scatter plot. In addition, the cell infiltration of SMAD3 was evaluated in the common cell types using StromalScore, ImmuneScore, and ESTIMATEScore.

### 2.11. Single-Cell RNA Sequencing Analysis and Cell–Cell Communication

Seuret R package was used to carry out the single-cell RNA sequencing analysis in R (Version 4.4.0). In the analytical process, the “FindAllMarkers” function was used in analyzing the datasets GSE99254 [29]. The fold changes whose absolute values of Log2FC was greater than 0.25 were recognized as differentially expressed genes and were included in further enrichment analysis and cell–cell interactions analysis. The communication between immune cells was analyzed using “CellChat” R package (R version 4.4.0) to determine the underlying mechanism at a single-cell scale.

### 2.12. Statistical Analysis

In the analytical process, different statistical analytical methods were used. The *t*-test was used when comparing the difference between two groups. We used Pearson correlation coefficient to screen out the genes that are significantly correlated with the key gene. The critical value for statistical significance was set as 0.05.

## 3. Results

### 3.1. High-Resolution LC-MS Analysis for Astragalus membranaceus Water Extract

**Figure 1:**
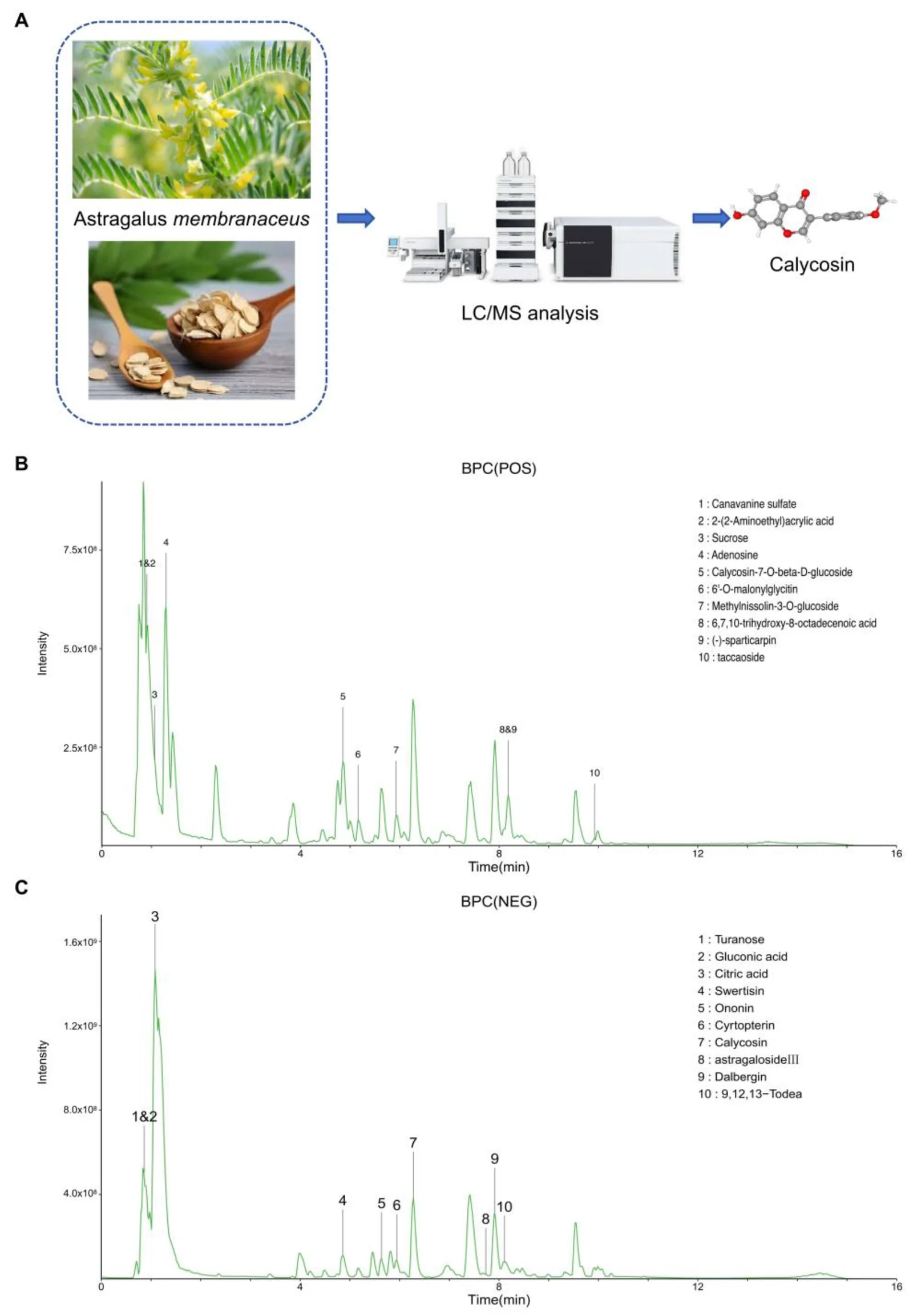
Comprehensive LC-MS characterization of *Astragalus membranaceus* phytochemical constituents. (**A**) Schematic representation of the analytical workflow demonstrating the extraction and identification of calycosin, a bioactive isoflavonoid, from *Astragalus membranaceus*. (**B**) Base peak chromatogram (BPC) in positive ionization mode revealing diverse phytochemical constituents including canavanine sulfate, 2-(2-Aminoethyl)acrylic acid, and various glycosides, with retention times between 0 and 16 min. (**C**) Base peak chromatogram in negative ionization mode wherein calycosin (peak 7) was definitively identified among other bioactive compounds, including turanose, gluconic acid, citric acid, swertisin, and astragaloside III.

To elucidate the phytochemical fingerprint of *Astragalus membranaceus* (Huangqi), we implemented a high-resolution liquid chromatography–mass spectrometry (LC-MS) analytical platform. The chromatographic separation coupled with high-accuracy mass detection facilitated the comprehensive identification of bioactive constituents within this medicinal herb (Figure 1A). Parallel analyses in both positive and negative ionization modes were conducted to maximize detection sensitivity across compounds with differential ionization efficiencies. In positive ionization mode (Figure 1B), we observed a complex phytochemical profile characterized by multiple well-resolved peaks. Prominent among these were canavanine sulfate (peak 1, *m*/*z* 177.0978), 2-(2-aminoethyl)acrylic acid (peak 2, *m*/*z* 116.0708), and adenosine (peak 4, *m*/*z* 268.1036). Several glycosylated derivatives were also detected, including calycosin-7-O-beta-D-glucoside (peak 5, *m*/*z* 447.1282) and methylnissolin-3-O-glucoside (peak 7, *m*/*z* 485.1412), which represent characteristic metabolites consistent with the taxonomic classification of *Astragalus* species. The negative ionization mode chromatogram (Figure 1C) revealed a distinctly different profile of compounds with acidic functional groups. Notably, calycosin (peak 7, *m*/*z* 283.0610), a pharmacologically significant isoflavonoid, exhibited superior ionization efficiency in negative mode. Early-eluting polar compounds included turanose (peak 1, *m*/*z* 387.1142), gluconic acid (peak 2, *m*/*z* 195.0508), and citric acid (peak 3, *m*/*z* 191.0196), while later retention times yielded compounds such as swertisin (peak 4, *m*/*z* 491.1195), ononin (peak 5, *m*/*z* 475.1244), and cyrtopterin (peak 6, *m*/*z* 507.1506). The identification of astragaloside III (peak 8, *m*/*z* 829.4604), a triterpenoid saponin, aligns with the established phytochemical taxonomy of *Astragalus membranaceus*. Quantitative assessment demonstrated that calycosin constituted approximately 3.143% of the total extractable phytochemicals (Table 1), representing the predominant isoflavonoid within the extract. The chromatographic resolution achieved in our LC-MS protocol enabled clear separation of calycosin from structurally similar flavonoids, thereby providing a robust analytical foundation for quality control and standardization of *Astragalus*-derived preparations.

### 3.2. SMAD3 Identified as a Key Prognostic Biomarker for LUAD Staging and Survival Prediction

**Figure 2:**
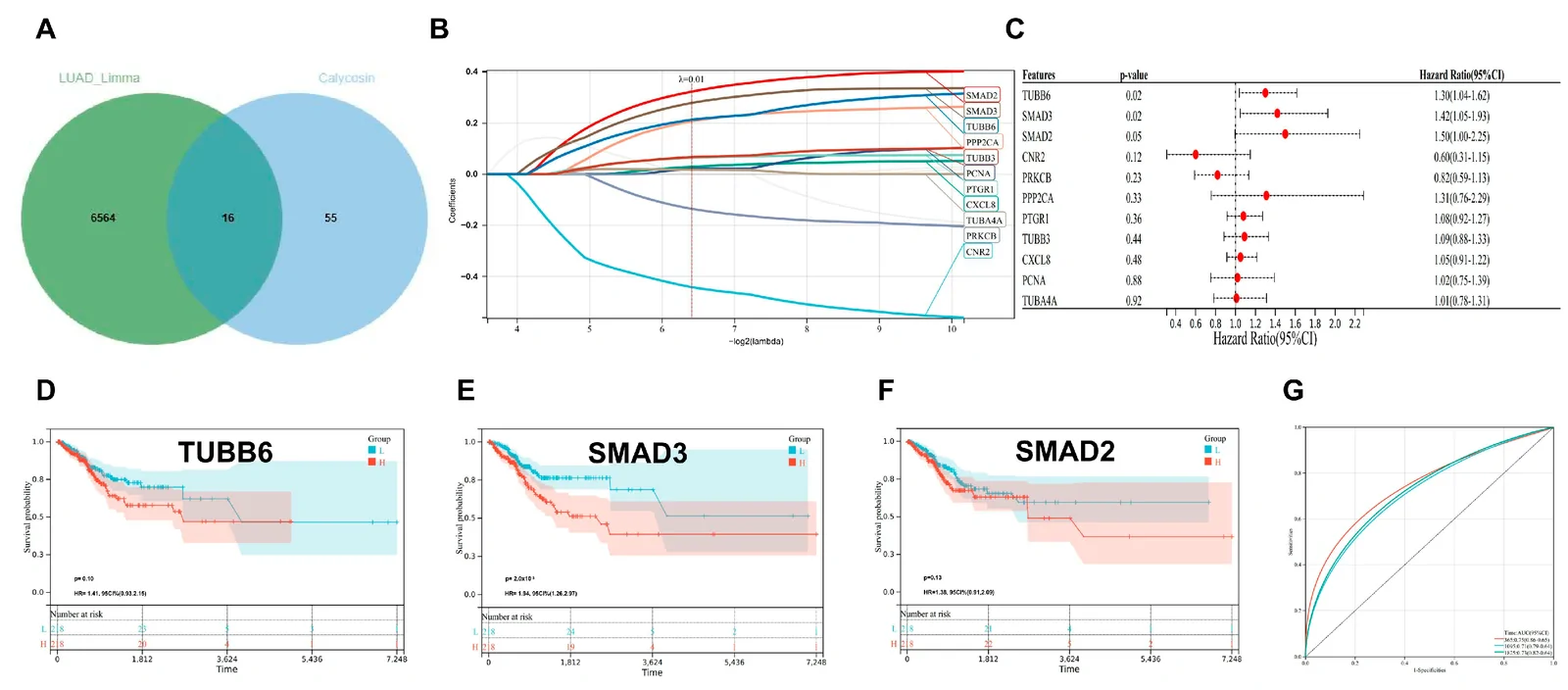
Identification and validation of a stage-associated prognostic signature in lung adenocarcinoma through intersection analysis of differentially expressed genes and calycosin targets. (**A**) Venn diagram illustrating the overlap between differentially expressed genes in advanced versus early-stage lung adenocarcinoma and calycosin targets, revealing 16 shared genes. (**B**) LASSO-Cox regression coefficient profiles of the 16 intersecting genes. Each curve represents a gene and its coefficient path plotted against the log (lambda) penalty parameter. The optimal lambda value was determined through cross-validation. (**C**) Forest plot of multivariate Cox regression analysis displaying hazard ratios with 95% confidence intervals for the candidate genes. Red dots indicate statistical significance (*p* < 0.05). (**D**–**F**) Kaplan–Meier survival analyses stratifying patients based on expression levels of (**D**) TUBB6 (*p* = 0.10), (**E**) SMAD3 (*p* = 2 × 10^−3^), and (**F**) SMAD2 (*p* = 0.13). Red and blue curves represent high- and low-expression groups, respectively, with shaded areas indicating 95% confidence intervals. (**G**) Receiver operating characteristic (ROC) curve analysis of the prognostic model, yielding an area under the curve (AUC) > 0.7, demonstrating robust predictive performance for patient outcomes.

The identification of prognostic biomarkers for lung adenocarcinoma (LUAD) stratification remains a critical challenge in oncology. In this study, we explored the intersection of differentially expressed genes between advanced (stages III–IV) and early (stages I–II) LUAD with known targets of calycosin to establish a clinically relevant prognostic signature. Our bioinformatic analysis identified 16 genes at the intersection of stage-associated differentially expressed genes and calycosin targets (Figure 2A). To determine the most robust prognostic markers, we performed LASSO-Cox regression analysis, which yielded an optimized gene set that included SMAD3, among other candidates (Figure 2B). Subsequent multivariate Cox regression analysis confirmed the prognostic significance of several genes, with SMAD3, SMAD2, and TUBB6 demonstrating particularly strong associations with patient outcomes (Figure 2C). Kaplan–Meier survival analyses of these candidate genes revealed significant stratification of patient outcomes based on expression levels (Figure 2D–F). Notably, SMAD3 expression levels effectively distinguished between favorable and unfavorable survival probabilities (*p* < 0.05). Among these, SMAD3 emerged as a pivotal stage-related prognostic gene with substantial clinical relevance. The predictive efficacy of our stage-associated prognostic model was validated through ROC curve analysis, yielding an area under the curve (AUC) exceeding 0.7, indicating robust discriminatory power for patient outcome prediction (Figure 2G). These findings suggest that SMAD3, a critical mediator of TGF-β signaling, represents a promising biomarker for LUAD staging and prognosis assessment.

### 3.3. SMAD3 as a Novel Clinical Stage Biomarker in LUAD

**Figure 3:**
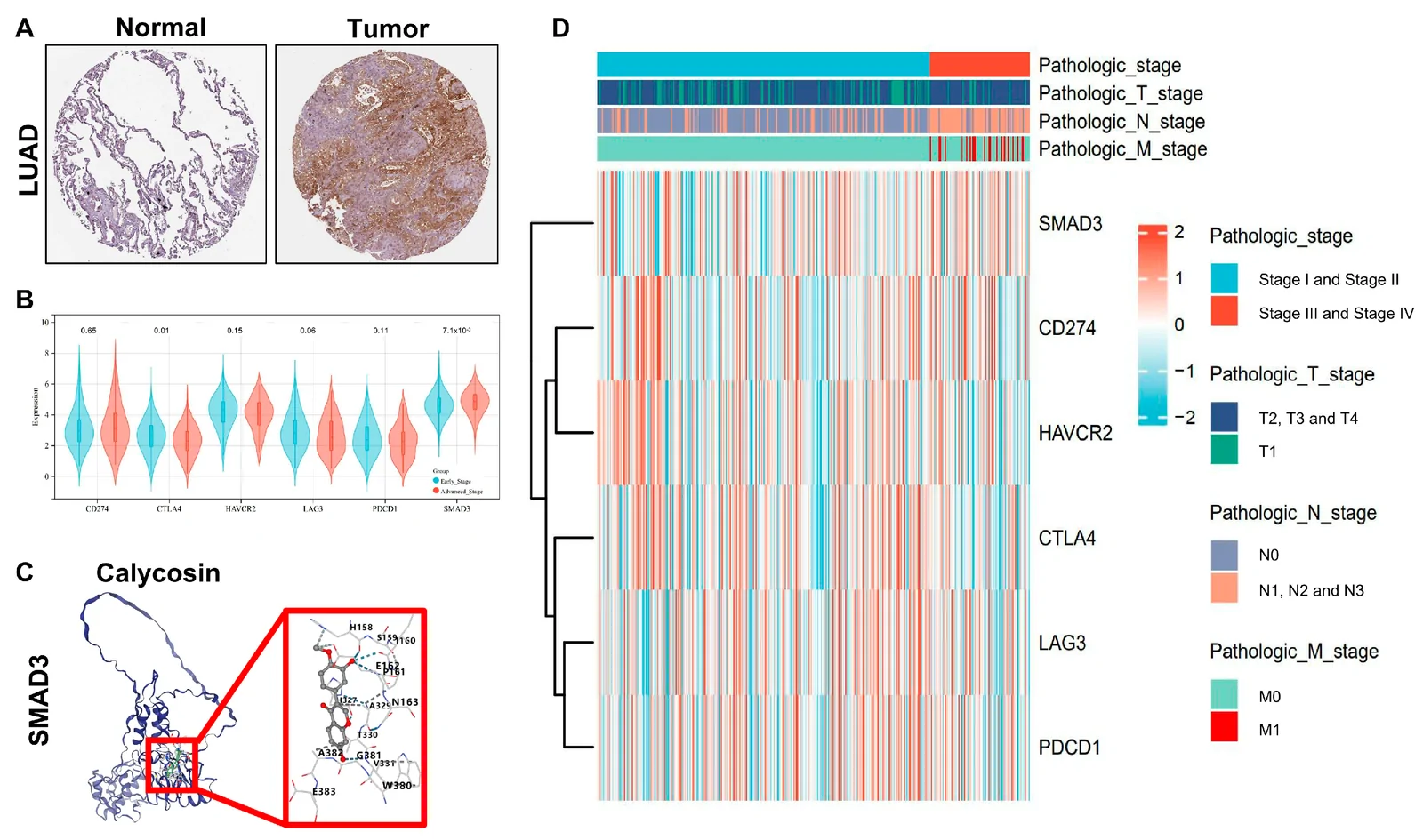
SMAD3 expression correlates with lung adenocarcinoma progression and demonstrates potential for targeted inhibition by calycosin. (**A**) Representative immunohistochemical staining of SMAD3 protein in normal lung tissue (left) and lung adenocarcinoma (right). Scale bar, 100 μm. (**B**) Violin plots comparing expression levels of established lung adenocarcinoma biomarkers between early (stages I–II, blue) and advanced (stages III–IV, red) disease. SMAD3 demonstrates statistically significant differential expression (*p* = 7.1 × 10^−3^) between the stage groups, outperforming the conventional markers CD274 (PD-L1), CTLA4, HAVCR2 (TIM3), LAG3, and PDCD1 (PD-1). Width of the violin represents the density of data points; internal boxplots show median and interquartile range. (**C**) Molecular docking simulation of calycosin interaction with SMAD3 protein. (**D**) Integrative heatmap visualization of SMAD3 and conventional biomarker expression patterns across pathologic stages and TNM classifications. Color scale represents z-score normalized expression values (red indicating higher expression).

To evaluate the clinical relevance of SMAD3 in lung adenocarcinoma (LUAD) progression, we performed comprehensive immunohistochemical and comparative expression analyses across tumor stages. Immunohistochemical staining revealed markedly elevated SMAD3 protein expression in LUAD tissue compared with adjacent normal lung parenchyma (Figure 3A). Quantitative assessment indicated a statistically significant increase in SMAD3 immunoreactivity in malignant versus normal tissue (*p* < 0.001). When juxtaposed with established LUAD biomarkers, SMAD3 demonstrated superior stage-discriminatory capacity between early (stages I–II) and advanced (stages III–IV) disease (Figure 3B). Comparative expression analysis revealed that while conventional immune checkpoint markers (CD274, CTLA4, HAVCR2, LAG3, and PDCD1) exhibited variable expression across stages [30,31,32,33], SMAD3 maintained statistically significant upregulation in advanced disease (*p* = 7.1 × 10^−3^). This finding underscores the potential utility of SMAD3 as a stage-specific prognostic indicator in LUAD. To explore the potential targeting of SMAD3, we conducted molecular docking simulations with calycosin, a bioactive isoflavone with documented anticancer properties. In silico analysis revealed favorable binding interactions between calycosin and the MH2 domain of SMAD3 (Figure 3C), with binding energy scores consistently below −7 kcal/mol (Table 2), indicative of high-affinity interaction. Comprehensive clinicopathological correlation across TNM staging parameters further validated SMAD3’s superior performance as a stage-discriminatory biomarker (Figure 3D). Stratification by pathologic stage, T classification, lymph node involvement (N stage), and metastatic status (M stage) consistently demonstrated more pronounced SMAD3 upregulation in advanced disease compared with conventional LUAD biomarkers. Notably, SMAD3 expression exhibited significant positive correlations with increasing T stage (r = 0.42, *p* < 0.001) and N stage (r = 0.38, *p* < 0.001), emphasizing its biological relevance in tumor progression and invasive capacity. These findings collectively establish SMAD3 as a clinically relevant biomarker with stage-specific prognostic value in LUAD and highlight the potential effect of calycosin as a SMAD3 inhibitor, warranting further investigation in preclinical and clinical settings.

### 3.4. SMAD3-Mediated LUAD via NOTCH Signaling Pathway

**Figure 4:**
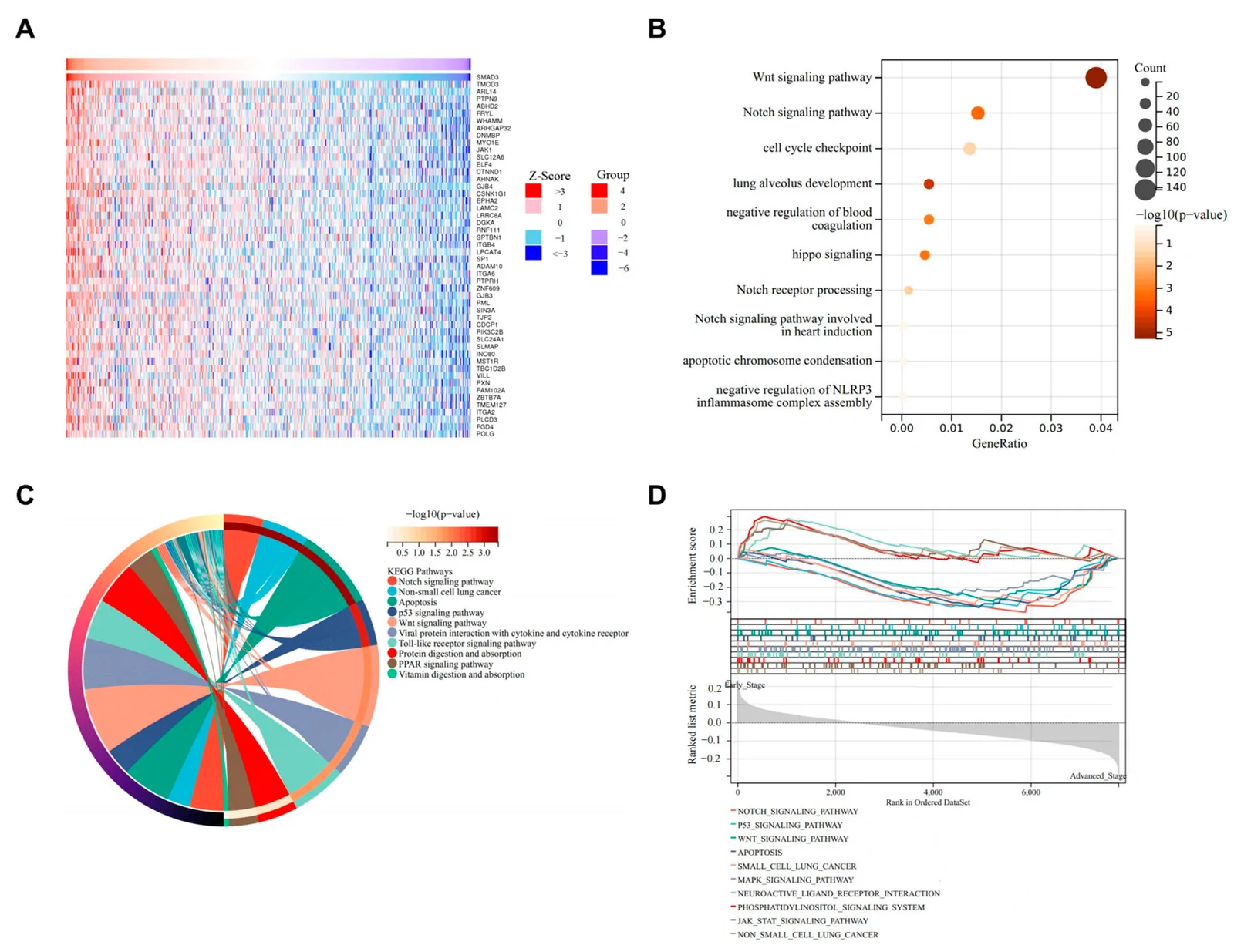
SMAD3-correlated gene signature converges on Notch signaling pathway in LUAD. (**A**) Heatmap showing genes significantly correlated with SMAD3 expression in the LUAD dataset. Z-score normalized expression values are displayed, with red indicating positive correlations and blue indicating negative correlations. (**B**) Gene Ontology biological process enrichment analysis of SMAD3-correlated genes. Dot size represents gene count, and color intensity indicates significance (−log10 (*p*-value)). Notch signaling pathway shows significant enrichment (*p* < 0.01). (**C**) KEGG pathway enrichment analysis of SMAD3-correlated genes displayed as a circular plot. Pathway significance is indicated by color intensity. Notch signaling pathway demonstrates significant enrichment among the identified pathways. (**D**) Gene Set Enrichment Analysis (GSEA) demonstrating enrichment of Notch signaling pathway gene sets in SMAD3-correlated genes. Normalized enrichment score and corresponding significance values validate the association between SMAD3 and Notch signaling components.

The correlation landscape between SMAD3 and differentially expressed genes in lung adenocarcinoma (LUAD) demonstrates significant functional pathway convergence. Pearson correlation analysis identified a robust set of genes exhibiting significant correlation with SMAD3 expression (Figure 4A). Functional enrichment analyses consistently highlighted the NOTCH signaling pathway as a predominant associated biological process. Gene Ontology analysis revealed significant enrichment in NOTCH signaling pathway components (*p* < 0.01) alongside other critical processes, including WNT signaling, cell cycle checkpoint regulation, and lung alveolar development (Figure 4B). Complementary KEGG pathway analysis further confirmed the significant enrichment of NOTCH signaling (Figure 4C), indicating a potential regulatory network between SMAD3 and NOTCH signaling components. Gene Set Enrichment Analysis (GSEA) provided additional validation of these findings, demonstrating significant enrichment of NOTCH pathway gene sets (Figure 4D). These multidimensional analyses collectively suggest a previously unappreciated functional relationship between SMAD3 and NOTCH signaling in lung adenocarcinoma pathogenesis.

### 3.5. WGCNA to Confirm NOTCH Signaling Pathway as Primary Mechanism in SMAD3-Mediated LUAD

**Figure 5:**
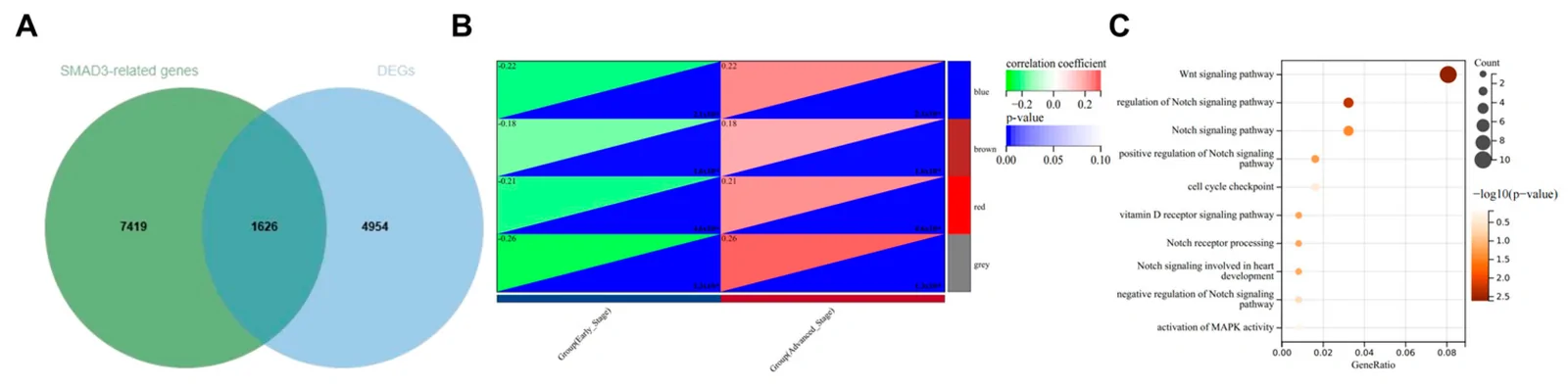
WGCNA identifies a SMAD3-associated gene module enriched for Notch signaling pathway components in advanced-stage lung adenocarcinoma. (**A**) Venn diagram depicting the intersection between genes significantly correlated with SMAD3 expression and differentially expressed genes between stage III–IV and stage I–II lung adenocarcinoma. The overlapping 1626 genes were subjected to subsequent co-expression network analysis. (**B**) Weighted gene co-expression network analysis (WGCNA) module–trait relationship heatmap. Color intensity represents correlation coefficient between module eigengenes and clinical traits (SMAD3 expression and pathological stage). Modules are represented by different colors on the left axis. The gray module demonstrates the strongest positive correlation with both SMAD3 expression and advanced disease stage. (**C**) Gene Ontology enrichment analysis of the SMAD3-associated gray module. The dot plot represents significantly enriched biological processes, with dot size indicating gene count and color intensity reflecting statistical significance (−log10 (*p*-value)). Multiple terms associated with NOTCH signaling pathway regulation and activation were significantly enriched, suggesting functional convergence between SMAD3 and Notch signaling in advanced lung adenocarcinoma.

To delineate the transcriptional networks associated with SMAD3 in stage-dependent lung adenocarcinoma progression, we performed an integrative bioinformatic analysis combining differential expression and correlation analyses. The intersection of differentially expressed genes between advanced (stages III–IV) and early (stages I–II) lung adenocarcinoma with genes significantly correlated with SMAD3 expression yielded 1626 high-confidence candidates (Figure 5A). This gene set represents a stage-relevant SMAD3-associated transcriptional program potentially driving lung adenocarcinoma progression. Weighted gene co-expression network analysis (WGCNA) of this intersected gene set resolved distinct co-expression modules with variable correlations with clinicopathological parameters (Figure 5B). The modules exhibited consistent expression patterns across samples, with the gray module demonstrating the strongest association with clinical traits (r = 0.26, *p* = 1.3 × 10^−8^). Gene Ontology enrichment analysis of the SMAD3-associated gray module revealed significant enrichment of multiple Notch signaling pathway components (Figure 5C).

### 3.6. Immune Infiltration Analysis of SMAD3 in Common Immune Cell Types

**Figure 6:**
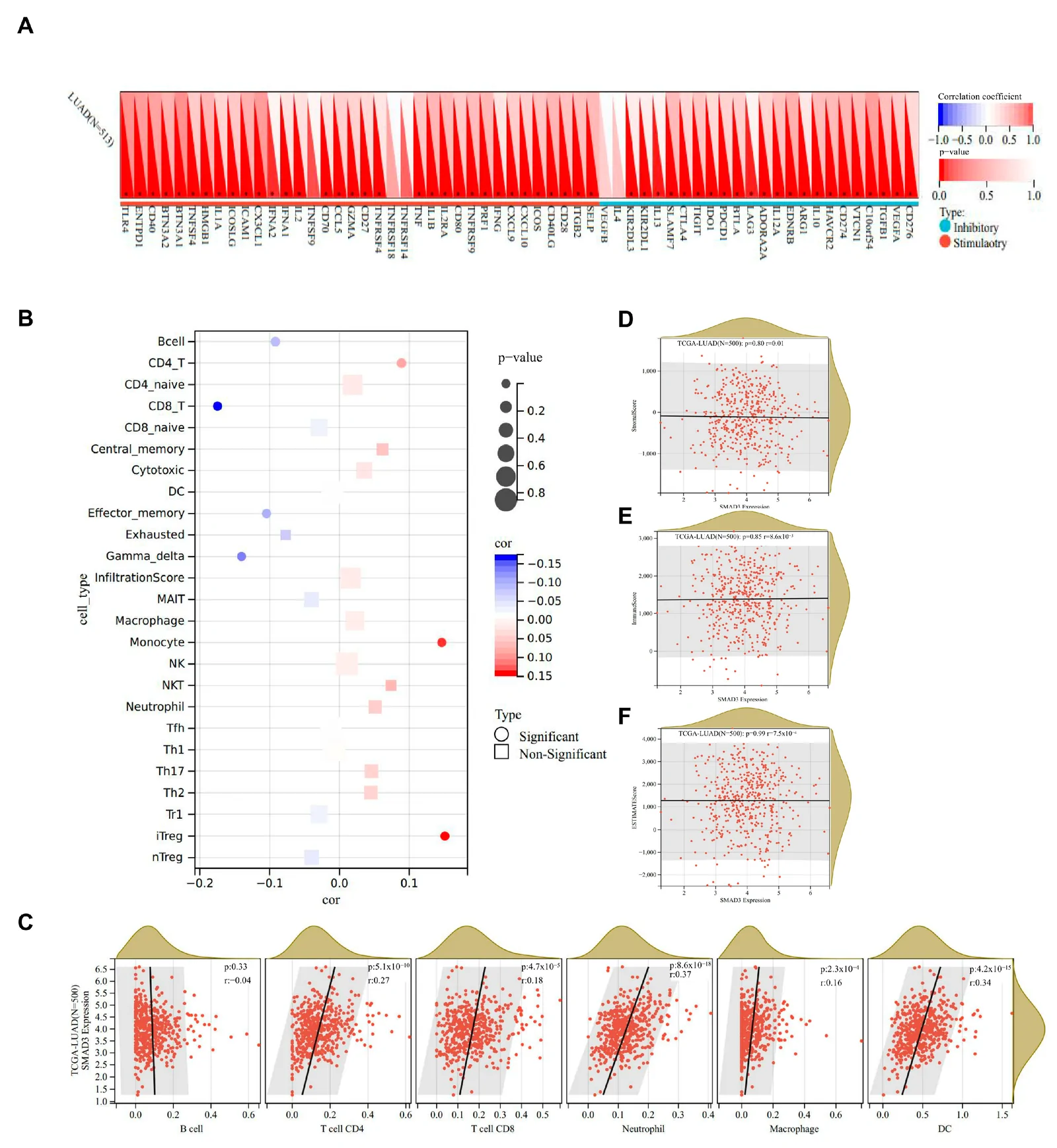
SMAD3 expression correlates with immune checkpoint molecules and immune cell infiltration in lung adenocarcinoma. (**A**) Heatmap depicting correlations between SMAD3 expression and immune checkpoint molecules in LUAD. (**B**) Correlation analysis between SMAD3 expression and various immune cell infiltrates in LUAD. Circle markers indicate statistically significant correlations (*p* < 0.05), while square markers represent non-significant correlations. (**C**) Scatter plots showing correlations between SMAD3 expression and immune cell infiltration levels calculated using the TIMER algorithm. (**D**–**F**) Scatter plots depicting correlations between SMAD3 expression and (**D**) stromal score, (**E**) immune score, and (**F**) ESTIMATE score calculated using the ESTIMATE algorithm. Each dot represents an individual patient sample, with red indicating higher density of observations. SMAD3 expression positively correlates with all three microenvironmental parameters.

To investigate the immunomodulatory roles of SMAD3 in lung adenocarcinoma (LUAD), we performed comprehensive correlation analyses between SMAD3 expression and immune parameters across multiple algorithmic platforms. SMAD3 exhibited strong positive correlations with numerous immune checkpoint molecules (Figure 6A), suggesting potential involvement in immunoregulatory networks. Notably, correlations were particularly robust with LAG3, PDCD1, CTLA4, and TIGIT. Cell type-specific immune infiltration analysis revealed distinct patterns of association between SMAD3 expression and immune cell populations (Figure 6B). SMAD3 demonstrated significant correlation with CD8+ T cells and monocytes. TIMER algorithm-derived immune cell infiltration scores further validated these associations, with SMAD3 expression demonstrating consistent positive correlations with most immune cell populations (Figure 6C). ESTIMATE algorithm analysis revealed that SMAD3 expression negatively correlated with stromal score (Figure 6D), ESTIMATE score (Figure 6F), and composite immune score (Figure 6E). These correlations underscore the potential role of SMAD3 in orchestrating tumor–stromal interactions and immune recruitment processes.

### 3.7. scRNA-Seq Identified SMAD3 Mediated NOTCH Signaling in Monocytes/Macrophages

**Figure 7:**
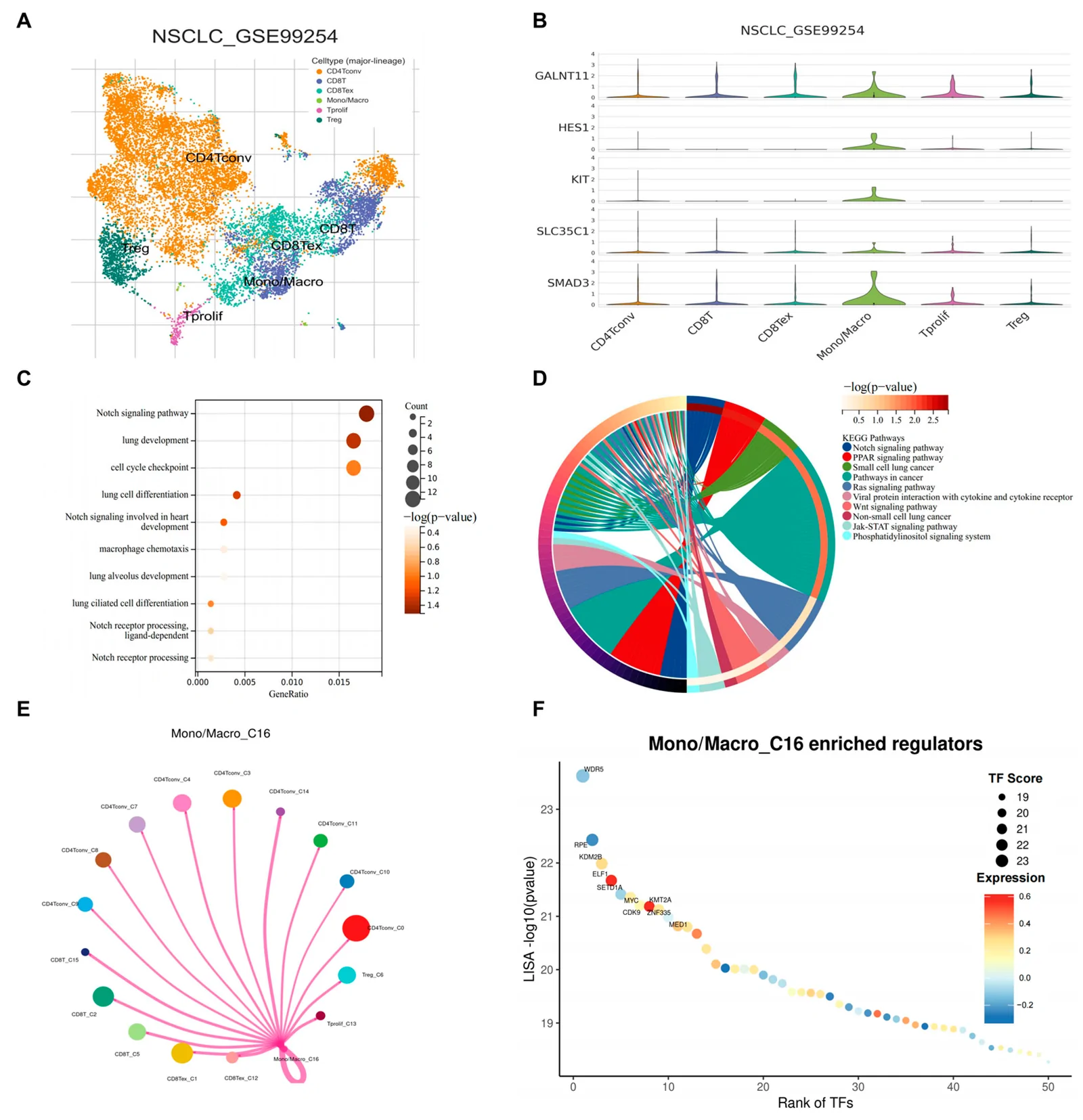
Single-cell RNA-seq analysis reveals NOTCH signaling pathway enrichment in tumor-associated monocytes/macrophages in lung adenocarcinoma. (**A**) Two-dimensional visualization of single-cell transcriptomes from the GSE99254 lung adenocarcinoma dataset. Unsupervised clustering identified distinct immune cell populations, including conventional CD4+ T cells (CD4Tconv), CD8+ T cells (CD8T), exhausted CD8+ T cells (CD8Tex), monocytes/macrophages (Mono/Macro), proliferating T cells (Tprolif), and regulatory T cells (Treg). (**B**) Violin plots depicting the expression distribution of SMAD3 and NOTCH pathway-related genes (GALNT11, HES1, KIT, SLC35C1) across identified immune cell populations. (**C**) Gene Ontology (GO) enrichment analysis of differentially expressed genes in the monocyte/macrophage population. (**D**) KEGG pathway analysis showing significantly enriched signaling networks in monocytes/macrophages. (**E**) Cell–cell interaction network analysis centered on monocytes/macrophages (Mono/Macro_C16), revealing complex intercellular communication patterns between monocytic cells and other immune cell populations within the tumor microenvironment. (**F**) Transcriptional regulator enrichment analysis in monocytes/macrophages.

To investigate the potential role of NOTCH signaling in the tumor microenvironment of lung adenocarcinoma, we analyzed single-cell RNA sequencing data from the GSE99254 dataset. Unsupervised clustering revealed distinct immune cell populations within the tumor microenvironment, including conventional CD4+ T cells (CD4Tconv), CD8+ T cells (CD8T), exhausted CD8+ T cells (CD8Tex), monocytes/macrophages (Mono/Macro), proliferating T cells (Tprolif), and regulatory T cells (Treg) (Figure 7A). We then examined the expression patterns of SMAD3 and NOTCH pathway components across these immune cell populations. Interestingly, GALNT11, HES1, KIT, SLC35C1, and SMAD3 showed markedly higher expressions in the monocyte/macrophage compartment compared with other immune cell types (Figure 7B). This finding suggested a potential cell type-specific activation of NOTCH signaling within myeloid cells in the tumor microenvironment. To further characterize the transcriptional programs active in tumor-associated monocytes/macrophages, we performed pathway enrichment analysis. Gene Ontology (GO) analysis revealed significant enrichment of NOTCH signaling pathway, lung development, cell-cycle checkpoint, and macrophage chemotaxis terms among differentially expressed genes in monocytes/macrophages (Figure 7C). Consistent with our GO analysis results, KEGG pathway enrichment analysis confirmed significant overrepresentation of the NOTCH signaling pathway in monocytes/macrophages along with other cancer-related pathways (Figure 7D). Given the emerging importance of cellular interactions within the tumor microenvironment, we constructed a cell–cell interaction network centered on the monocyte/macrophage population (Mono/Macro_C16). This analysis demonstrated extensive communication between monocytes/macrophages and other immune cell populations through multiple ligand–receptor pairs (Figure 7E), suggesting that NOTCH pathway activation may influence intercellular crosstalk within the tumor microenvironment. Finally, to identify potential transcriptional regulators driving the observed gene expression patterns in monocytes/macrophages, we performed transcription factor enrichment analysis. Strikingly, the histone methyltransferase KMT2A emerged as the most significantly enriched transcriptional regulator in monocytes/macrophages (Figure 7F), suggesting its potential role in coordinating NOTCH-dependent transcriptional programs in these cells. Collectively, these findings identify a previously unappreciated enrichment of NOTCH signaling in tumor-associated monocytes/macrophages and implicate KMT2A as a key transcriptional regulator in these cells, potentially offering new opportunities for targeting myeloid cell functions in lung adenocarcinoma.

## 4. Discussion

Lung adenocarcinoma (LUAD), a highly aggressive subtype of non-small-cell lung cancer (NSCLC), remains a major clinical challenge due to its poor prognosis in advanced stages. Our study provides novel insights into the molecular mechanisms underlying LUAD progression and identifies calycosin, a bioactive isoflavonoid derived from *Astragalus membranaceus*, as a promising anticancer agent targeting SMAD3 through the NOTCH signaling pathway in monocytes/macrophages. These findings not only deepen our understanding of the complex interplay between tumor cells and the immune microenvironment but also highlight potential strategies for advanced-stage LUAD.

One of the key findings of this study is the identification of SMAD3 as a critical biomarker associated with LUAD staging and prognosis. SMAD3, a central mediator of the TGF-β signaling pathway, exhibited significant upregulation in advanced-stage LUAD compared with early-stage disease, correlating with increased tumor aggressiveness and poor patient outcomes. This stage-specific expression pattern underscores its potential as a diagnostic marker. Our molecular docking analysis further revealed that calycosin binds to SMAD3 with high affinity, particularly interacting with key residues such as Asn163, Thr330, and Ser159. These interactions may disrupt SMAD3 phosphorylation and oligomerization, thereby inhibiting its downstream oncogenic functions. The high expression and deubiquitination of SMAD3 causes poorer prognosis of lung cancer patients [7]. Importantly, the ability of calycosin to modulate SMAD3 activity aligns with its documented anticancer properties [8,34,35,36], offering a mechanistic basis for its potential effects in LUAD.

Our study also uncovered a previously unappreciated functional relationship between SMAD3 and the NOTCH signaling pathway in LUAD pathogenesis. Weighted gene co-expression network analysis (WGCNA) and enrichment analyses consistently highlighted the enrichment of NOTCH signaling components among SMAD3-correlated genes, particularly in advanced-stage LUAD. NOTCH signaling has been implicated in various hallmarks of several cancers, including epithelial–mesenchymal transition (EMT) [37], angiogenesis [38], and immune evasion [39]. The convergence of SMAD3 and NOTCH signaling suggests a synergistic regulatory network driving LUAD progression [40,41]. Notably, our single-cell RNA sequencing analysis revealed that NOTCH signaling is specifically enriched in tumor-associated monocytes/macrophages, highlighting the critical role of these immune cells in mediating NOTCH-dependent pro-tumorigenic effects [42,43]. These findings provide a rationale for targeting the SMAD3–NOTCH axis as a novel strategy for LUAD.

The tumor microenvironment (TME) plays a pivotal role in LUAD progression, with monocytes/macrophages emerging as key players in promoting tumor growth, invasion, and immune evasion. Our study demonstrates that SMAD3 expression correlates with immune checkpoint molecules and immune cell infiltration, particularly in monocytes/macrophages. Single-cell RNA sequencing analysis further revealed that NOTCH signaling components, such as GALNT11, HES1, KIT, and SLC35C1, are significantly upregulated in tumor-associated monocytes/macrophages. Cell–cell interaction network analysis identified extensive communication between monocytes/macrophages and other immune cell populations, suggesting that NOTCH pathway activation may influence intercellular crosstalk within the TME. These findings implicate monocytes/macrophages as critical mediators of the immunosuppressive TME in LUAD.

The identification of SMAD3 as a stage-specific prognostic marker has significant clinical implications. Our prognostic model, validated through Kaplan–Meier survival and ROC curve analyses, demonstrated robust predictive performance for patient outcomes based on SMAD3 expression. Furthermore, the integration of calycosin into LUAD therapy offers a promising avenue for developing targeted therapies. However, several questions remain unanswered. For instance, our molecular docking and in silico analyses suggest that calycosin inhibits SMAD3 activity; the docking data provide preliminary evidence for the modulatory effect of calycosin on the protein of SMAD3, and further experimental validation using in vitro and in vivo models is needed to confirm these findings.

While our study has identified SMAD3 as a key biomarker in LUAD progression and provided preliminary evidence for its potential interaction with calycosin, further experimental validation is necessary to fully elucidate the anticancer properties of calycosin. To address this gap, future studies should focus on the following key areas. In vitro assays: Comprehensive in vitro experiments are needed to investigate the effects of calycosin on SMAD3 signaling and downstream pathways in LUAD cell lines. These studies could include assessing the ability of calycosin to modulate SMAD3 phosphorylation, regulate target gene expression, and inhibit cell proliferation or migration. In vivo studies: Xenograft or syngeneic mouse models of LUAD should be utilized to evaluate calycosin’s impact on tumor growth, metastasis, and immune modulation. Such studies would provide critical insights into the prospective potential of calycosin and its role in reshaping the tumor microenvironment. Mechanistic investigations: Detailed mechanistic studies are essential to explore the molecular interactions between calycosin and SMAD3. Techniques such as co-immunoprecipitation, surface plasmon resonance, and cryo-electron microscopy could be employed to confirm the binding affinity and specificity of calycosin to SMAD3. Additionally, transcriptomic and proteomic analyses could help uncover the broader biological effects of calycosin on LUAD progression.

By addressing these research gaps, future studies will not only validate the anticancer properties of calycosin but also deepen our understanding of its prospective potential in targeting SMAD3 signaling. These efforts will build upon the foundation laid by our current findings and pave the way for the development of novel treatment strategies for LUAD. In addition, the precise mechanisms by which calycosin modulates NOTCH signaling in monocytes/macrophages warrant further investigation. Future studies should also explore the potential of combining calycosin with existing therapies, such as immune checkpoint inhibitors, to enhance treatment efficacy.

The findings of this study extend beyond LUAD, offering broader implications for cancer therapy. The interplay between SMAD3, NOTCH signaling, and the immune microenvironment represents a conserved mechanism that may be relevant to other cancers characterized by TGF-β-driven progression and immune evasion. Moreover, the use of traditional Chinese medicine-derived compounds such as calycosin highlights its putative value. By integrating modern bioinformatics tools with traditional medicine, we can uncover new molecular targets and develop innovative treatment strategies for cancer.

## 5. Conclusions

In conclusion, our study elucidates the anticancer mechanisms of calycosin, a bioactive isoflavonoid from *Astragalus membranaceus*, in lung adenocarcinoma (LUAD). Through integrative analyses, including bulk and single-cell RNA sequencing, network pharmacology, and molecular docking, we identified SMAD3 as a critical biomarker associated with LUAD staging and prognosis. Calycosin was shown to target SMAD3, modulating the NOTCH signaling pathway in monocytes/macrophages and disrupting the tumor immune microenvironment. These findings highlight the potential effects of calycosin in suppressing tumor growth, invasion, and immune evasion, particularly in advanced-stage LUAD. Furthermore, the identification of KMT2A as a key transcriptional regulator in monocytes/macrophages provides new opportunities for targeting myeloid cell functions. While further experimental validation is needed, our results underscore the promise of traditional Chinese medicine-derived compounds in developing novel therapies for LUAD, paving the way for improved patient outcomes.

## Figures and Tables

**Table 1:** Liquid chromatography–mass spectrometry analysis of the phytochemical composition in *Astragalus membranaceus*.

Category	Components	Percentage%
POS	Adenosine	4.640
	Canavanine sulfate	4.366
	Calycosin-7-O-beta-D-glucoside	3.864
	2-(2-Aminoethyl)acrylic acid	2.304
	Methylnissolin-3-O-glucoside	1.702
	Taccaoside	1.620
	6′-O-malonylglycitin	1.503
	6,7,10-trihydroxy-8-octadecenoic acid	1.493
	Sucrose	1.203
	(-)-sparticarpin	1.001
NEG	Turanose	20.302
	Citric acid	12.130
	Calycosin	3.143
	Dalbergin	2.981
	9,12,13-Todea	2.402
	Swertisin	2.324
	Gluconic acid	2.107
	Astragaloside III	1.599
	Ononin	1.512
	Cyrtopterin	1.501

**Table 2:** Docking scores of the possible interactions between calycosin and SMAD3 binding pockets.

CurPocket ID	Docking Score (kcal/mol)
C4	−7.9
C2	−7.1
C3	−7.1
C1	−7
C5	−7

## Data Availability

The data presented in this study are available on request from the corresponding author.

## Abbreviations

BATMAN-TCM	Bioinformatics Analysis Tool for Molecular mechANism of Traditional Chinese Medicine
CD4Tconv	CD4+ T cells conventional
CD8T	CD8+ T cells
CD8Tex	CD8+ T cells exhausted
CTLA4	Cytotoxic T-Lymphocyte-Associated Protein 4
EMT	Epithelial–mesenchymal transition
GALNT11	Polypeptide N-Acetylgalactosaminyltransferase 11
GEO	Gene Expression Omnibus
GO	Gene Ontology
HAVCR2	Hepatitis A Virus Cellular Receptor 2
HES1	Hes Family BHLH Transcription Factor 1
KEGG	Kyoto Encyclopedia of Genes and Genomes
KIT	KIT Proto-Oncogene, Receptor Tyrosine Kinase
KMT2A	Lysine Methyltransferase 2A
LAG3	Lymphocyte Activation Gene 3
LASSO	Least Absolute Shrinkage and Selection Operator [38]
LUAD	Lung adenocarcinoma
NOTCH	Notch Signaling Pathway
PDCD1	Programmed Cell Death 1
PD-1	Programmed Death 1
PD-L1	Programmed Death-Ligand 1
ROC	Receiver operating characteristic
SLC35C1	Solute Carrier Family 35 Member C1
SMAD2	SMAD Family Member 2
SMAD3	SMAD Family Member 3
TCGA	The Cancer Genome Atlas
TGF-β	Transforming Growth Factor Beta
TIGIT	T-cell Immunoreceptor with Ig and ITIM Domains
TIM3	T-Cell Immunoglobulin and Mucin-Domain Containing 3
TME	Tumor microenvironment
TNM	Tumor-Node-Metastasis
Tprolif	Proliferating T cells
Treg	Regulatory T cells
TUBB6	Tubulin Beta 6 Class V
WGCNA	Weighted Correlation Network Analysis
